# Efficacy of cationic polymer-coated magnesium oxide nanoparticles as anti-cancer candidates

**DOI:** 10.1098/rsos.250656

**Published:** 2025-07-09

**Authors:** Muhammad Hunain Shahid, Maninder Singh, Robin Rajan, Kazuaki Matsumura

**Affiliations:** ^1^ Japan Advanced Institute of Science and Technology (JAIST), Nomi, Ishikawa, Japan

**Keywords:** MgO nanoparticles, cationic polymers, nanocomposites, anti-cancer candidates, *in vitro* cytotoxicity

## Abstract

Conventional cancer therapies are frequently limited by systemic toxicity and inadequate selectivity, necessitating the development of novel therapeutic approaches. Nanotechnology has emerged as a promising platform for achieving targeted and drug-free cancer treatments. In this study, we report a drug-free anti-cancer strategy based on cationic polymer-coated magnesium oxide nanoparticles (MgO NPs). The nanocomposites (NCs) were fabricated by grafting cationic 3-acrylamidopropyl trimethyl ammonium chloride (AMPTMA)-based polymers onto 3-aminopropyltriethoxysilane (APTES)-functionalized MgO NPs, thereby integrating the cytotoxic properties of MgO, potentially mediated by reactive oxygen species (ROS) generation and Mg²^+^ ion release, with the membrane-targeting capacity of cationic polymers. Cytotoxicity assessments indicated that commercial MgO NPs exhibited minimal anti-cancer activity. While both poly-AMPTMA (PAMPTMA) homopolymer and its copolymer (PAMPTMA-*r*-BuMA) demonstrated potent cytotoxicity, they lacked selectivity, affecting both cancerous and normal cells. In contrast, the polymer-modified MgO NCs markedly enhanced cytotoxicity against cancer cell lines (A-549 half-maximal inhibitory concentrations (IC_50_): 202 and 64 µg ml^−1^; Colon-26 IC_50_: 338 and 115 µg ml^−1^) while reducing toxicity towards normal cells (human dermal fibroblast (HDF) IC_50_: 180 and 226 µg ml^−1^). Notably, the MgO-APTES-PAMPTMA-*r*-BuMA nanocomposite exhibited superior selectivity and efficacy, presumably through enhanced membrane disruption and ROS production. These findings underscore the potential of polymer-functionalized MgO NCs as a promising drug-free anti-cancer platform and contribute to the advancement of nanomedicine-based therapeutics.

## 1. Introduction

Cancer remains the second leading cause of mortality worldwide, following cardiovascular diseases, with nearly 20 million new cases and approximately 10 million deaths reported in 2022, according to the World Health Organization [1]. Despite advancements in cancer therapies, conventional approaches, including chemotherapy, radiation and immunotherapy, face significant limitations, such as systemic toxicity, limited tumour specificity, low bioavailability, rapid clearance and inconsistent efficacy [2,3]. These challenges underscore the urgent need for a deeper understanding of cancer pathogenesis and the development of innovative therapeutic strategies. Nanomaterials have emerged as promising candidates in cancer treatment, offering enhanced therapeutic outcomes and reduced side effects through passive and active targeting strategies [4]. Among various nanomaterials, metal nanoparticles (MNPs), distinguished by their unique physico-chemical properties, hold significant potential for biomedical applications, including biosensors, drug delivery systems, photo-ablation and hyperthermia therapy [5]. Metal and metal oxide nanoparticles, such as Pd, Au, Cu, Pt, Fe_3_O_4_, MgO and Cu_2_O, address many challenges associated with conventional chemotherapy by exhibiting tumour-specific accumulation through the enhanced permeability and retention (EPR) effect, enabling targeted drug delivery and modulating the tumour microenvironment [6–9].

Magnesium oxide nanoparticles (MgO NPs), known for their excellent functionalization and biodegradable properties, have gained attention as promising therapeutic agents in applications such as antibacterial treatments, cardiovascular therapies and bone regeneration [10,11]. Their anti-cancer potential arises from their ability to generate reactive oxygen species (ROS) and release Mg^2+^ ions, which induce cytotoxicity in cancer cells [12,13]. For instance, Di *et al.* demonstrated that biodegradable MgO NPs enhance cryosurgical efficacy by improving thermal conductivity and promoting ice formation, resulting in more effective tumour cell destruction [14]. However, challenges such as nanoparticle aggregation, improper metal ion distribution and instability within cellular environments can limit their efficacy and lead to unintended cytotoxicity. These challenges can be addressed through surface modification with ligands, polar functional groups, stabilizing agents or embedding the nanoparticles in a polymer matrix as composites, enhancing their biomedical potential [15,16]. Among these strategies, nanocomposite formation stands out as a simple and effective approach to prevent MNP aggregation while enhancing their functional properties.

Polymers and their nanocomposites (PNCs) are widely utilized in contemporary biomedicine due to their exceptional biocompatibility, biodegradability, cytotoxicity and functionality [17,18]. PNCs, consisting of functional polymers and nanoparticle fillers, exhibit enhanced properties by synergistically integrating the unique physico-chemical characteristics of their components. Polymer–metal nanocomposites (PMNCs) have garnered significant attention for their dual diagnostic and therapeutic capabilities, providing low toxicity and highly efficient solutions for cancer treatment [15,19]. Alfaro *et al.* developed MgO-PEG-2ME nanocarriers for the targeted delivery of 2-methoxy estradiol (2ME), achieving a drug-loading efficiency of 98.51% with sustained release over 7 days across varying pH levels. *In vitro* studies demonstrated a 40% reduction in prostate cancer (LNCap) cell viability within 72 h, showcasing their potential as an effective drug delivery platform [20]. Polymers embedded with MNPs have significantly contributed to the advancement of nanocomposites (NCs) by preventing nanoparticle aggregation and preserving functional properties. These formulations exhibit improved stability and biocompatibility, making them highly effective for targeted therapy, imaging and cancer treatment [21,22].

Synthetic polymer matrices, such as polyethylene glycol (PEG) and polylactic acid (PLA), have gained substantial attention in NCs for their stability, flexibility, biodegradability and low immunogenicity [23]. Furthermore, the potential of polymers as stand-alone anti-cancer agents is an emerging and promising area of research. Cationic polymers, in particular, exhibit anti-cancer potential through electrostatic interactions with anionic cancer cell membranes, which are attributed to the overexpression of anionic lipids. This interaction ultimately induces cell lysis [24,25]. Cationic polymers have also demonstrated efficacy in combating tumour metastasis [26], overcoming drug resistance [27], exhibiting antimicrobial properties [28] and targeting dormant cancer cells [29]. The quaternary ammonium chloride monomer, 3-acrylamidopropyl trimethyl ammonium chloride (AMPTMA), characterized by its inherent positive charge, has been applied in drug delivery systems, microcarriers for cell proliferation and gene delivery [30,31]. Recently, Kumar *et al.* synthesized novel anti-cancer cationic homopolymers of AMPTMA and its copolymers with hydrophobic moieties, enhancing their anti-cancer activity against various cancer cell lines [32]. However, while cationic polymers with hydrophobic groups have shown promise in anti-cancer applications, their selective cytotoxicity and potential as nanocomposite formulations with MNPs remain unexplored. Despite the individual potential of MgO NPs and cationic polymers like AMPTMA in cancer therapy, comprehensive studies on their combined synergistic efficacy in NC formulations are lacking.

To address this gap, we developed novel NCs by integrating anti-cancer polymers with hydrophobic–cationic chains onto functionalized MgO NPs to create a drug-free therapeutic platform. The cationic polymer AMPTMA and its random copolymer with butyl methacrylate (BuMA) as a hydrophobic co-monomer were synthesized via reversible addition–fragmentation chain transfer (RAFT) polymerization. By varying the BuMA content, polymer compositions were tailored to investigate the influence of hydrophobicity on anti-cancer efficacy. To enhance dispersion, stability and amine group functionalization, MgO NPs were modified with the silane coupling agent of 3-aminopropyltriethoxysilane (APTES) through a reflux condensation method. The NC formulations were achieved by grafting the synthesized polymers onto functionalized MgO NP surfaces via amide bond formation. The anti-cancer potential of the synthesized NCs, bare NPs and polymers was systematically evaluated against cancer and normal cell lines using cell viability assays. This study aims to achieve a synergistic approach by combining cationic polymers with hydrophobic domains and functionalized MgO NPs to create a drug-free nanocomposite platform capable of selectively targeting and eliminating cancer cells while reducing toxicity to healthy tissues. By leveraging the dual functionality of cationic polymers for cancer cell membrane disruption and MgO NPs for potential cytotoxicity, these NCs offer a promising alternative to conventional chemotherapy. The findings could pave the way for next-generation nanomedicine, providing a targeted therapeutic approach with enhanced efficacy and reduced systemic side effects.

## 2. Experimental section

### 2.1. Materials

Magnesium oxide nanopowder (Brunauer−Emmett−Teller (BET) size less than or equal to 50 nm, white colour), 3-aminopropyl triethoxysilane (APTES; coupling agent), 2-(dodecylthiocarbonothioylthio)-2-methyl propanoic acid *N*-hydroxy-succinimide ester (RAFT agent) and azobisisobutyronitrile (AIBN; initiator) were procured from Sigma-Aldrich Chemical Company (Saint Louis, MO, USA). A 75% aqueous solution of the monomer (3-acrylamidopropyl) trimethylammonium chloride (AMPTMA), hydrogen peroxide (H_2_O_2_; oxidizing agent), *n*-butyl methacrylate (BuMA; hydrophobic co-monomer) and ninhydrin reagent were purchased from TCI (Tokyo Chemical Industry, Japan). BuMA was purified before use by passing it through a packed column to remove the inhibitor (inhibitor removers; Sigma-Aldrich). Diethylene dioxide (1,4-dioxane) was obtained from FUJIFILM Wako Pure Chemical Corporation. Deuterium oxide (D_2_O) was sourced from Thermo Scientific Chemicals. The MTT reagent (3-(4,5-dimethyl-2-thiazolyl)-2,5-diphenyl-2H-tetrazolium bromide) was supplied by DOJINDO Laboratories Corporation Limited. For *in vitro* cell culture studies, Dulbecco’s modified Eagle’s medium (DMEM) supplemented with 10% fetal bovine serum (FBS) was obtained from Nacalai Tesque, Inc. (Japan). The Colon-26 (mouse colon adenocarcinoma), A-549 (human lung adenocarcinoma) and human dermal fibroblast (HDF) cell lines used in this study were acquired from the American Type Culture Collection (ATCC) for cytotoxicity assessments.

### 2.2. Synthesis

#### 2.2.1. Functionalization of magnesium oxide nanoparticles

The surface functionalization of MgO NPs was conducted using APTES in ethanol (EtOH) solvent, following a modified protocol based on Chandran *et al.* [33]. A reflux condensation method was employed for the functionalization. Initially, commercially available MgO NPs were oxidized with H_2_O_2_ to enhance the hydroxyl (–OH) groups on the nanoparticle surface, facilitating the subsequent condensation reaction with APTES. Approximately 4.5 g of MgO NPs were dispersed in 750 ml of H_2_O_2_ (6 mg ml^−1^) in a round-bottom flask and sonicated for 24 h in a sonication bath. After oxidation, the MgO NPs were thoroughly washed three to four times with deionized water (DI) and collected by centrifugation at 8000 r.p.m. for 10 min. The washed NPs were then dried at 60°C for 24 h. Subsequently, the oxidized MgO NPs were dispersed in a mixture of EtOH and DI water (95 : 5 v/v) in a round-bottom flask using a sonication bath to prepare them for the surface condensation reaction. Once properly dispersed, 2 ml of APTES was added to the mixture, and the reaction was carried out at 80°C for 24 h in an oil bath equipped with a reflux condenser. After the reaction, the functionalized MgO NPs were washed twice with DI water and EtOH at 8000 r.p.m. for 10 min to remove unreacted APTES and other impurities. The functionalized MgO-APTES NPs were dried overnight in a vacuum oven at 60°C. These NPs were subsequently characterized and incorporated into PNC formulations to evaluate their cytotoxicity against cancer and normal cell lines.

#### 2.2.2. Synthesis of cationic polymers

Cationic polymers, PAMPTMA_100_ (homopolymer) and PAMPTMA_80_-*r*-BuMA_20_ (random copolymer), were synthesized using RAFT polymerization, following a modified protocol by Kumar *et al.* [32]. For PAMPTMA_100_, AMPTMA monomers (14.5 mmol, 3 g), RAFT agent (0.145 mmol, 66.99 mg) and AIBN (0.02 mmol, 4.76 mg) were combined in a molar feed ratio of AMPTMA : RAFT agent: AIBN = 100 : 1 : 0.2 and dissolved in a dioxane–water mixture (7 : 3 v/v). For PAMPTMA_80_-*r*-BuMA_20_, AMPTMA (19.35 mmol, 4 g), BuMA (4.83 mmol, 686.82 mg), RAFT agent (0.193 mmol, 89.33 mg) and AIBN (0.038 mmol, 6.35 mg) were combined in a molar feed ratio of AMPTMA : BuMA : RAFT agent : AIBN = 80 : 20 : 1 : 0.2 and dissolved in the same solvent mixture. In both cases, the reaction mixtures were purged with nitrogen gas for 30−40 min to eliminate oxygen, sealed with a rubber septum and stirred at 75°C for 24 h. The resulting polymers were purified by dialysis against distilled water for 4 days using a 4k−6k MWCO Spectra/Por dialysis membrane and subsequently lyophilized. The synthesized homopolymer and copolymer were characterized and utilized for NCs formulation, with their cytotoxicity evaluated against cancer and normal cell lines.

#### 2.2.3. Formation of nanocomposites

**Scheme 1.**
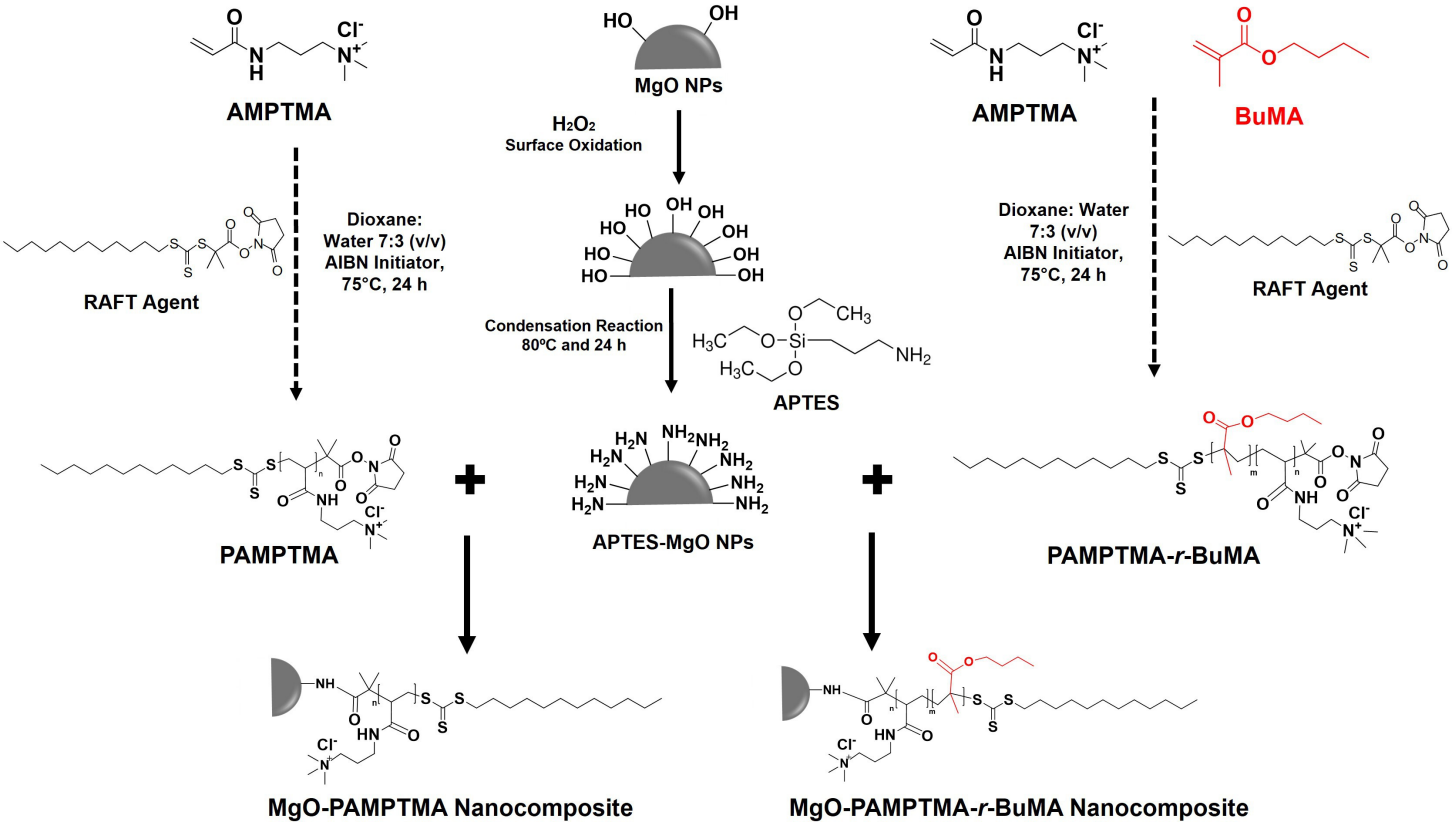
Reaction scheme for the synthesis of MgO nanocomposites.

NCs were synthesized by grafting PAMPTMA (homopolymer) and PAMPTMA-*r*-BuMA (copolymer) onto APTES-functionalized MgO NPs (Scheme 1). For both NCs, 250 mg of APTES-functionalized MgO NPs were dispersed in phosphate-buffered saline (PBS, pH 7.4) and subjected to sonication for 60 min to ensure uniform dispersion. Separately, 500 mg of each polymer (PAMPTMA or PAMPTMA-*r*-BuMA) was dissolved in PBS (pH 7.4) using a vortex mixer to prepare a polymer solution. The polymer solution was gradually introduced into the MgO NPs dispersion under continuous stirring, maintaining a nanoparticle-to-polymer weight ratio of 1 : 2 (w/w) to achieve optimal grafting density. The reaction mixtures were stirred continuously at room temperature for 24 h to allow effective polymer grafting onto the nanoparticle surfaces. Subsequently, the mixtures were centrifuged and washed with DI and EtOH four times to remove unreacted polymers and other impurities. The final NC products were dried in a vacuum oven at 50°C for 24 h. The synthesized NCs were subjected to detailed characterization, and their cytotoxicity was evaluated against various cancer and normal cell lines.

### 2.3. Characterization

The synthesized cationic polymers, functionalized MgO NPs and NCs were characterized using several advanced analytical techniques to confirm their structural, chemical and thermal properties.

#### 2.3.1. X-ray diffraction

The crystalline structures of bare MgO NPs, APTES-functionalized MgO NPs and NCs were examined using a Rigaku Mini-Flex600 diffractometer with a Cu Kα radiation source (λ = 1.5406 Å). Operating at 40 kV and 15 mA, the instrument provided detailed insights into the crystalline phases of the samples.

#### 2.3.2. Transmission electron microscopy

The morphology of MgO NPs was examined using transmission electron microscopy (TEM) on a Hitachi H-7650 microscope. The sample was prepared by drop-casting on a carbon-coated copper TEM grid using a micropipette of a dilute solution of EtOH, and subsequently allowed to air dry at room temperature. High-resolution images were obtained to observe particle shape and size.

#### 2.3.3. Scanning electron microscopy and energy dispersive X-ray spectroscopy

The surface morphology and elemental composition of MgO NPs, APTES-functionalized MgO NPs and NCs were investigated using desktop scanning electron microscopy (SEM) (TM3030 Plus, Hitachi) equipped with energy dispersive X-ray (EDS) capabilities. This analysis confirmed the functionalization process and provided elemental mapping of the samples.

#### 2.3.4. Fourier-transform infrared spectroscopy

Fourier-transform infrared spectroscopy (FTIR) was conducted to identify surface functional groups and confirm chemical modifications. Measurements were performed using a PerkinElmer Spectrum 100 spectrometer in attenuated total reflectance (ATR) mode with a diamond/KRS-5 crystal. Spectra were recorded over the range of 4000−400 cm⁻¹ with 32 accumulated scans, ensuring precise identification of functional groups introduced during modification and grafting.

#### 2.3.5. X-ray photoelectron spectroscopy

X-ray photoelectron spectroscopy (XPS) was used to analyse the surface chemical composition of the samples. Measurements were conducted on a Fisons S-probe TM 2803 instrument with a monochromatic Cu K*α* radiation source, providing detailed information on elemental states and chemical bonding on the nanoparticle surfaces.

#### 2.3.6. Thermogravimetric analysis

Thermal stability and decomposition behaviour of the samples were evaluated using thermogravimetric analysis (TGA) on a Rigaku TG 8121 Thermo plus EV02 instrument. Samples were heated from room temperature to 800°C at a rate of 10°C min^−1^ under a nitrogen atmosphere to avoid oxidation.

#### 2.3.7. Dynamic light scattering and zeta potential

Dynamic light scattering (DLS) and zeta potential measurements were conducted using a Zeta Sizer Nano-ZS 3000 (Malvern Instruments, UK). These tests assessed the size distribution and surface charge of MgO NPs, functionalized MgO NPs, cationic polymers and NCs. Measurements were performed using disposable cuvettes and folded capillary cells (DTS-1070) at a scattering angle of 173°, providing insights into the dispersion stability and surface properties of the materials.

#### 2.3.8. Ninhydrin test

A qualitative ninhydrin test was conducted to confirm the presence of NH_2_ groups on APTES-functionalized MgO NPs. A 1% ninhydrin solution was added to pristine MgO NPs, APTES-MgO NPs and NCs. The appearance of Ruhemann’s purple colour in APTES-MgO NPs confirmed successful surface modification, while no colour change was observed for pristine MgO NPs, and only faint coloration was detected for NCs.

#### 2.3.9. Nuclear magnetic resonance

The chemical structure of the synthesized cationic polymers was verified using proton nuclear magnetic resonance (¹H-NMR) spectroscopy. Spectra were recorded on a Bruker Avance III NEO 400 spectrometer operating at 400 MHz with 250 scans. Chemical shifts were referenced to the solvent peak at *δ* = 4.79 ppm for D_2_O.

#### 2.3.10. Gel permeation chromatography

The molecular weight and polydispersity index (PDI) of the synthesized polymers were determined using a Waters e2695 gel permeation chromatography (GPC) system equipped with an Ultra-hydrogel 250 column and a 2414 refractive index detector. A 10% methanol-PBS solution (pH 7.4) was used as the mobile phase at a flow rate of 1 ml min^−1^. Calibration was performed using the Pullulan Shodex standards.

### 2.4. Cytotoxicity determination

#### 2.4.1. Cell Culture

Colon-26 (mouse colon adenocarcinoma), A-549 (human lung carcinoma) and HDF cell lines were obtained from the ATCC. The cells were cultured in DMEM supplemented with 10% FBS and incubated at 37°C in a humidified atmosphere containing 5% CO_2_. Upon reaching confluence, the cells were rinsed with PBS to remove residual media and detached using a trypsin–EDTA solution (0.25% [w/v] trypsin with 0.02% [w/v] EDTA in PBS). Detached cells were collected, resuspended in fresh DMEM and seeded into new culture plates for subculturing. This protocol ensured the availability of viable and proliferative cells for subsequent *in vitro* cytotoxicity studies.

#### 2.4.2. 3-(4,5-Dimethylthiazol-2-yl)-2,5-diphenyltetrazolium bromide assay

The cytotoxic effects of bare MgO NPs, cationic polymers and NCs were evaluated against cancer cells (A-549, Colon-26) and normal cells (HDF) using the 3-(4,5-dimethylthiazol-2-yl)-2,5-diphenyltetrazolium bromide (MTT) assay. Cells were seeded at a density of 1 × 10⁴ cells per well in 100 µl of culture medium in a 96-well plate. The cells were cultured in DMEM supplemented with 10% FBS and incubated for 24 h at 37°C in a humidified atmosphere containing 5% CO_2_. Following incubation, the medium was replaced with fresh DMEM containing varying concentrations of MgO NPs, polymers and NCs, and cells were incubated for 48 h under the same conditions. After the treatment, 100 µl of MTT solution (300 µg ml^−1^ in DMEM) was added to each well, followed by a 4 h incubation to allow the formation of formazan crystals. The MTT solution was then carefully removed, and 100 µl of dimethyl sulfoxide (DMSO) was added to each well to dissolve the formazan crystals. Absorbance was measured at 570 nm using a microplate reader (Infinite 200Pro, Infinite M Nano, Tecan). All experiments were conducted in triplicate to ensure the reproducibility and reliability of the results.

### 2.5. Statistical analysis

All data are presented as the mean ± standard deviation (s.d.), based on triplicate experiments. Statistical comparisons were performed using two-way ANOVA followed by Tukey’s multiple comparison test, with statistical significance set at *p* < 0.05.

## 3. Results and discussion

### 3.1. Polymer characterization

**Figure 1.**
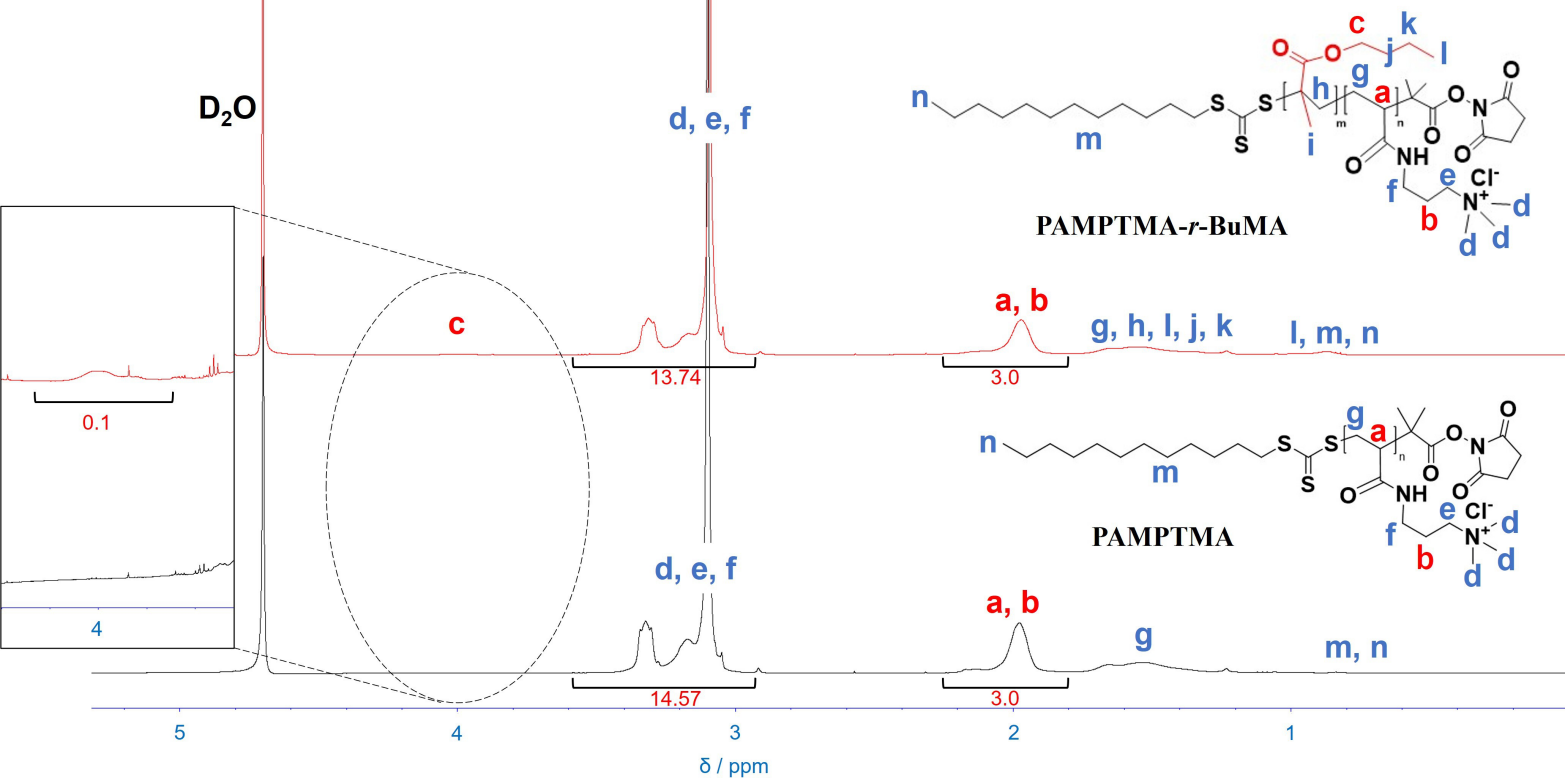
^1^H-NMR spectra (400 MHz, D_2_O) of homopolymer AMPTMA and copolymer AMPTMA-*r*-BuMA.

The homopolymer (PAMPTMA) and random copolymer (PAMPTMA-*r*-BuMA) were characterized using ¹H-NMR, confirming successful polymer synthesis via RAFT polymerization. The disappearance of the monomer vinyl protons (*δ* = 5.0−5.5 ppm) in the spectra provided evidence for polymerization. PAMPTMA-*r*-BuMA was synthesized by incorporating BuMA at a molar feed ratio of AMPTMA : BuMA = 80 : 20. The ¹H-NMR spectrum of PAMPTMA displayed characteristic signals corresponding to its structural components. A broad peak at *δ* = 3.2 ppm was attributed to the methylene protons (CH_2_) adjacent to the quaternary ammonium group (N^+^(CH_3_)_3_). The multiplets at (*δ* = 2.0 ppm) were assigned to the methylene protons (CH_2_) further along the polymer backbone, while the broader signal at (*δ* = 1.4 ppm) corresponded to other methylene protons within the polymer backbone. In addition, a singlet at *δ* = 3.1 ppm was identified as the methyl protons (CH_3_) of the quaternary ammonium group (figure 1). In the case of PAMPTMA-*r*-BuMA, the spectrum exhibited all characteristic peaks of PAMPTMA, along with distinct new signals attributed to the BuMA moiety. A prominent peak at *δ* = 4.0−4.2 ppm was assigned to the methylene protons (OCH_2_) of the BuMA ester group, confirming successful copolymerization (figure 1). By varying the feed ratio of BuMA during synthesis, its incorporation into the polymer was controlled. The degree of BuMA incorporation was quantified by comparing the integral of the methylene signal from BuMA (*δ* = 4.0−4.2 ppm) with that of the methylene protons from AMPTMA (*δ* = 1.8−2.3 ppm). The relatively low BuMA incorporation rate is probably due to solvent effects, steric hindrance, reactivity ratios and reaction conditions (table 1). Despite these challenges, the successful introduction of BuMA into the polymer structure highlights the potential for tailoring polymer properties by modulating monomer feed ratios.

**Table 1.** Characteristics of synthesized cationic polymers.

polymers		composition		ζ (mV)	M_n_ × 10^4 b^	PDI (M_w_/M_n_)^b^
		AMPTMA	BuMA			
PAMPTMA	in-feed in polymer^a^	100	0	57.3 ± 4.78	2.5	1.3
PAMPTMA-*r*-BuMA	in-feed in polymer^a^	80 94.3	20 5.7	59.7 ± 5.63	1.6	1.2

^a^ Determined by ^1^H-NMR.

^b^ Determined by GPC.

GPC revealed that the PAMPTMA homopolymer had an average molecular weight (M_n_) of 2.5 × 10⁴ g mol^−1^ and a PDI of 1.3, indicating a moderately broad molecular weight distribution. The incorporation of 20% BuMA during copolymerization reduced the molecular weight of PAMPTMA-*r*-BuMA to 1.6 × 10⁴ g mol^−1^ and narrowed the PDI to 1.2, indicating improved molecular weight distribution (table 1). The decrease in molecular weight and PDI reflects the influence of BuMA on polymerization kinetics and chain termination. These results align well with reported values for similar RAFT polymerization systems, as highlighted in studies by Kumar *et al.*, demonstrating consistent control over molecular weight and distribution [32]. We also calculated the zeta potential of homopolymer and copolymer, which showed positive zeta potential values in ultrapure water (UPW), attributed to the quaternary ammonium groups in the PAMPTMA backbone. These positive zeta potentials indicate strong electrostatic repulsion between particles, contributing to colloidal stability and minimizing aggregation (table 1). Furthermore, both polymers demonstrated miscibility in cell culture media (DMEM with and without FBS) and water.

### 3.2. Nanoparticle and nanocomposite characterization

The MgO NPs procured from Sigma-Aldrich were characterized to confirm their physico-chemical properties. The MgO NPs, appearing as pure white powder, were determined to have a size less then or equal to 50 nm and a specific surface area of 50−80 m² g^−1^ as measured by BET analysis. TEM analysis revealed that the MgO NPs predominantly exhibit cuboidal morphology, with an average particle size of 20−40 nm (electronic supplementary material, figure S1). EDS analysis confirmed the elemental composition, showing only magnesium (Mg) and oxygen (O₂), with no detectable impurities. The detailed physico-chemical characteristics of the MgO NPs are summarized in table 2.

**Table 2.** Properties of magnesium oxide nanoparticles in powder form.

physico-chemical properties of MgO NPs	
appearance	white powder
BET size	≤50 nm
specific surface area	50−80 m^2^ g^−1^
TEM size and shape	approximately 20–40 nm, mostly cuboidal
EDS	no impurities
X-ray diffraction	conforms to structure

XRD analysis revealed the crystalline and structural changes in MgO NPs induced by surface functionalization and NCs formation. The bare MgO NPs exhibited sharp diffraction peaks at 2θ values of 36.7°, 42.3°, 62.3°, 74.6° and 78.6°, corresponding to the face-centred cubic (FCC) structure with Miller indices (111), (200), (220), (311) and (222). These results were consistent with PDF 00-004-0829, confirming the high purity and crystallinity of the MgO NPs. The average crystallite size was calculated to be 12.4 nm using the Debye–Scherrer equation. Functionalization with APTES introduced additional peaks (approx. 50.2° and 53.7°), a shift in the diffraction peak from 42.3° to 37.1° and broadening of peaks, resulting in a crystallite size reduction to 4.02 nm. These changes suggest surface strain, defect formation and partial amorphization caused by APTES intercalation, which also improved nanoparticle dispersion. Similar results have been reported by Alkadhem *et al.* [34]. The NCs (both homopolymer and copolymer) showed further peak broadening and reduced intensity compared with bare and functionalized NPs, with a crystallite size reduction to 3.91 nm due to the incorporation of polymers (PAMPTMA and PAMPTMA-*r*-BuMA). The incorporation of polymers increased the amorphous content, strain and disorder within the MgO crystallites while retaining the cubic structure (electronic supplementary material, figure S2).

**Figure 2.**
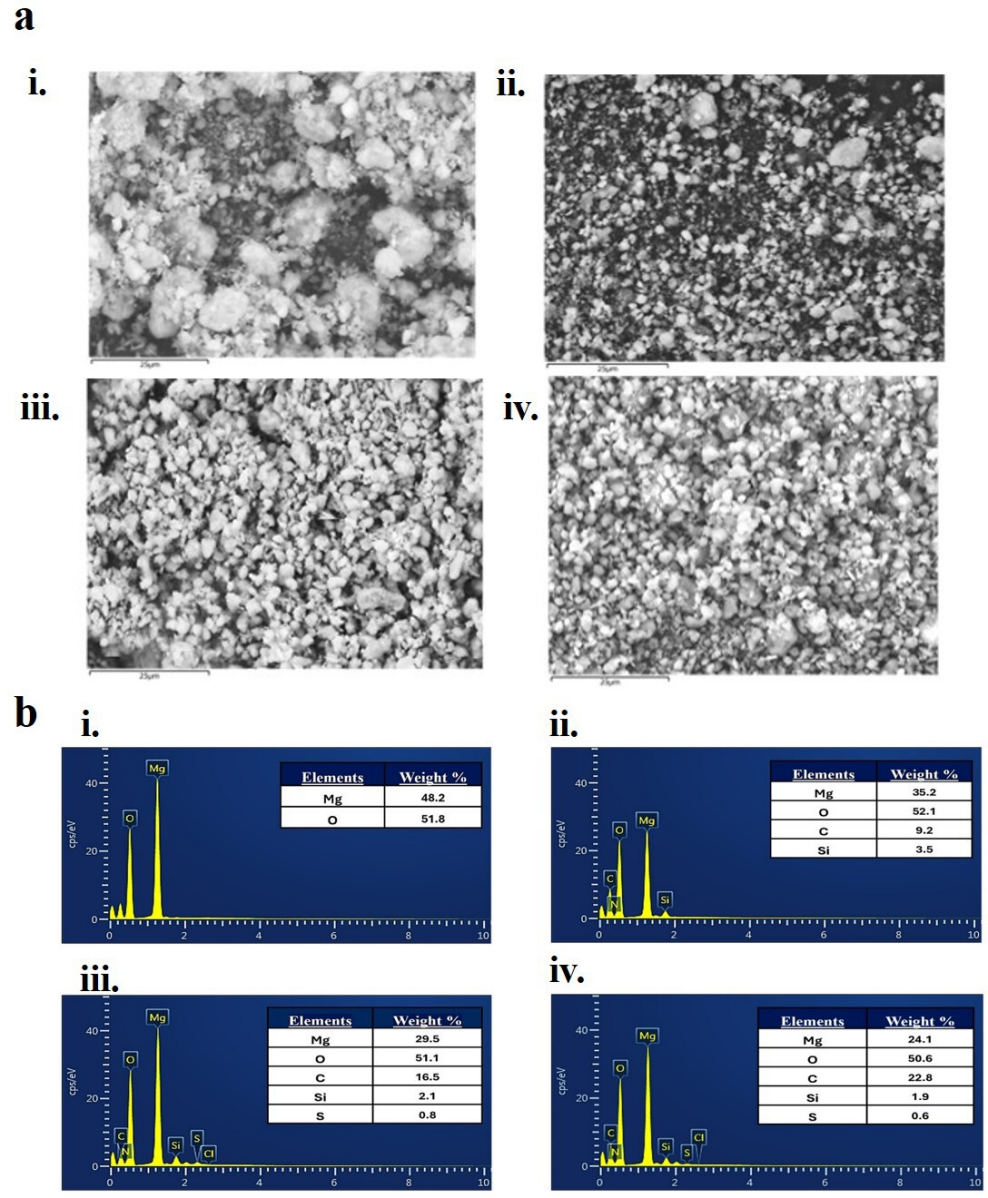
(a) Surface morphologies (scale bar: 25 µm) and (b) elemental compositions with SEM-EDS (10 keV) of (i) MgO NPs, (ii) functionalized MgO NPs, (iii) nanocomposite homopolymer and (iv) nanocomposite copolymer.

The surface morphology, composition and elemental mapping of MgO NPs and NCs were analysed using SEM coupled with EDS (figure 2 and electronic supplementary material, figure S3). SEM images of MgO NPs showed agglomerated structures with irregular shapes and non-uniform sizes due to high surface energy. The EDS spectrum confirmed high purity, with prominent peaks for Mg and O_2_ and no significant impurities. Upon functionalization with APTES, SEM images revealed smoother surfaces, indicating effective coating, while EDS analysis detected additional peaks for silicon (Si), carbon (C) and nitrogen (N), confirming successful functionalization [35]. For the NCs, SEM images showed uniform morphologies and densely packed structures with reduced aggregation, demonstrating successful polymer integration with functionalized NPs. The homopolymer-grafted NCs exhibited homogeneously distributed NPs within the polymer matrix, while the copolymer-grafted NCs displayed interconnected and intricate structures (figure 2a). EDS spectra of both NCs revealed peaks for Mg, O, Si, C, N, sulfur (S) and chlorine (Cl). The prominent carbon peak indicated the presence of the polymer backbone. Peaks for Si and N confirmed the presence of APTES, while S and Cl peaks were attributed to residual components from the polymerization process, including S/Cl ions from the PAMPTMA polymer (figure 2b). Elemental mapping demonstrated uniform element distribution, highlighting the homogeneous incorporation of NPs within the polymer matrices (electronic supplementary material, figure S3). These findings are consistent with those reported by Chandran *et al.* [33] for NCs incorporating polyvinylidene fluoride and ZnO-APTES.

**Figure 3.**
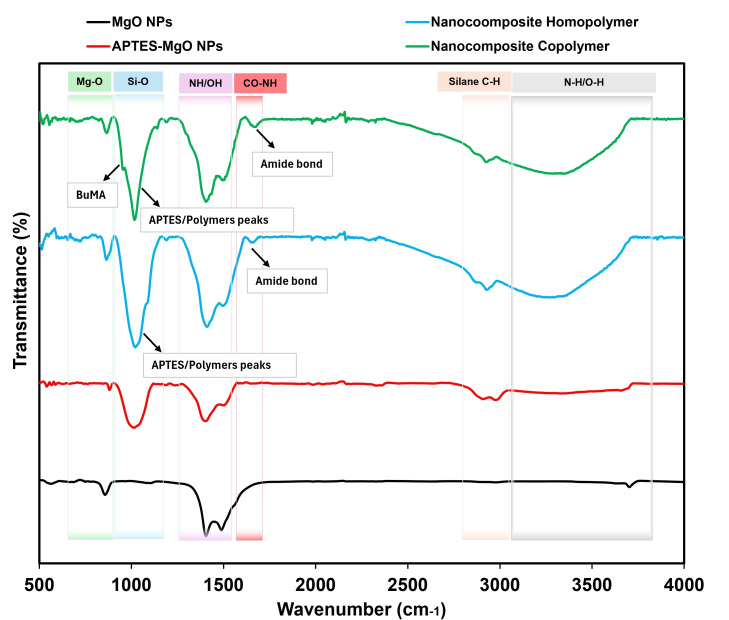
FTIR spectra of MgO NPs, APTES functionalized MgO NPs and NCs.

FTIR, TGA and XPS were employed to confirm the surface modifications that occurred. Figure 3 shows the FTIR transmittance spectra of all samples in the wavenumber range of 400–4000 cm⁻¹. The FTIR spectrum of bare MgO NPs exhibited characteristic metal–oxygen vibrations with prominent peaks at approximately 565 and 850 cm⁻¹, corresponding to Mg–O stretching. Peaks at 1400–1488 and 3700 cm⁻¹ indicated the presence of unoxidized hydroxyl (–OH) groups absorbed moisture [36]. Following APTES functionalization, new peaks emerged in the FTIR spectrum of MgO NPs. Notable peaks included 1073 cm⁻¹ (Si–O–Mg stretching), 2904 and 2971 cm⁻¹ (C–H stretching) and 1560 cm⁻¹ (N–H bending), confirming the successful attachment of APTES functional groups [34,35]. The FTIR spectra of NCs revealed absorption bands characteristic of both APTES-functionalized MgO NPs and the polymers. Distinct peaks included 1652 cm⁻¹ (C=O stretching from the polymer backbone) and a broad band at 3000–3700 cm⁻¹ (N–H and O–H stretching and bending). The FTIR spectrum of PAMPTMA in the NCs showed additional peaks characteristic of the polymer, such as quaternary ammonium group vibrations at 1470 cm⁻¹, N–H stretching at 1540 cm⁻¹ and C=O stretching at 1630 cm⁻¹, as reported by Mendonça *et al.* [37]. For the NC-copolymer, a peak at 1088 cm⁻¹ was attributed to C–O–C stretching from the BuMA co-monomer, indicating successful copolymer incorporation. The appearance of characteristic amide peaks further confirmed the successful integration of PAMPTMA and PAMPTMA-*r*-BuMA onto functionalized MgO NPs. However, the quaternary ammonium peaks were not distinctly resolved in the NC spectra due to overlapping signals from hydroxyl groups in MgO NPs.

**Figure 4.**
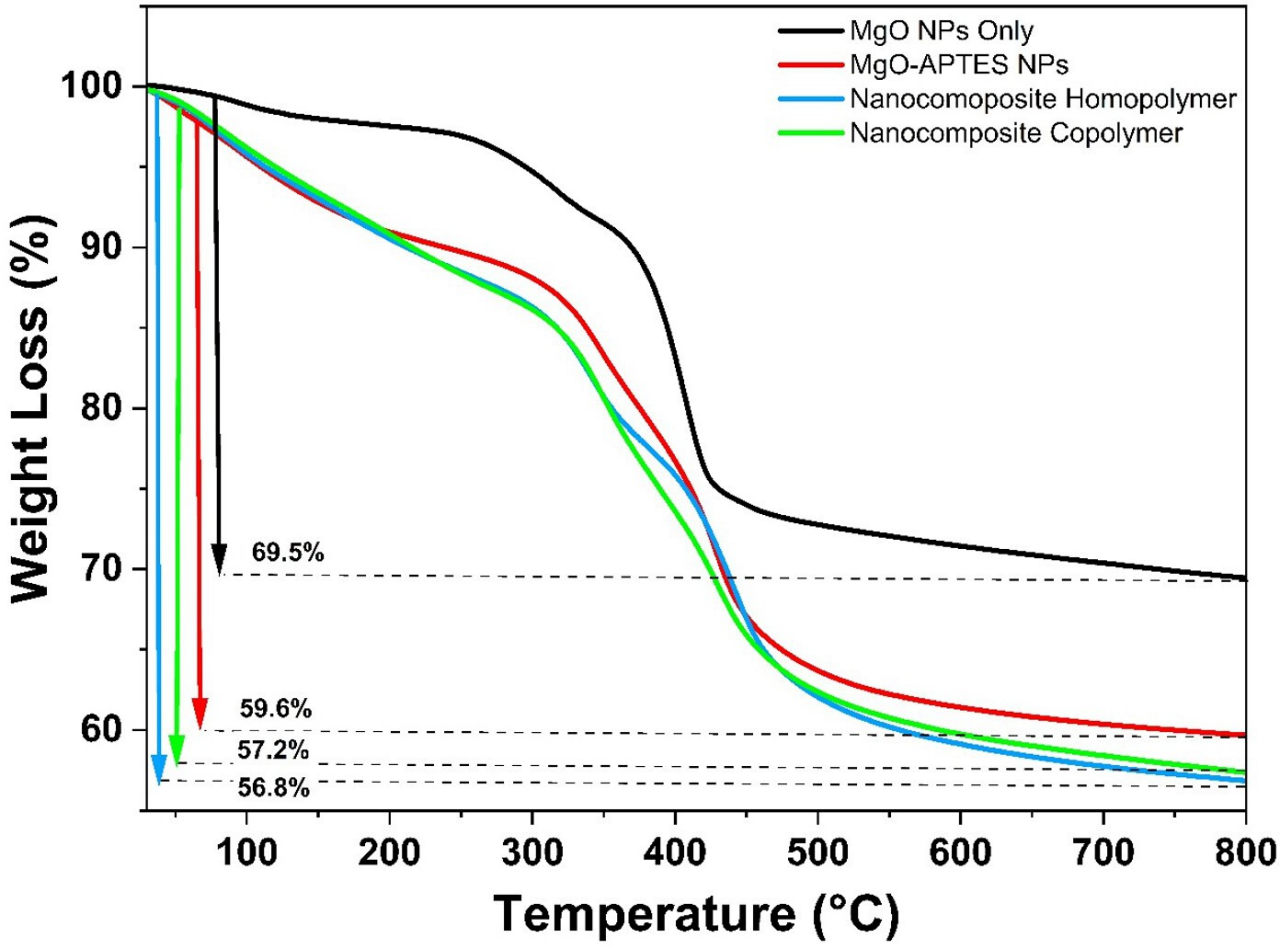
TGA plots of MgO NPs, APTES functionalized MgO NPs and NCs.

TGA was employed to evaluate the thermal stability and surface modifications of pristine MgO NPs, APTES-functionalized NPs and NCs. Figure 4 illustrates the TGA curves of the analysed samples, showing weight loss associated with thermal decomposition processes [38]. The TGA curve of bare MgO NPs exhibited significant weight loss (69.5% at 800°C), primarily due to the removal of adsorbed water molecules and surface hydroxyl groups. For APTES-functionalized MgO NPs, weight loss occurred in two stages: 50–250°C, attributed to the hydrolysis of residual ethoxy groups and physically adsorbed APTES, and 250–800°C, corresponding to the chemical degradation of covalently bonded APTES molecules, indicating successful functionalization. For NCs, the degradation behaviour reflected the thermal properties of the polymers. Initial weight loss up to 200°C was due to moisture evaporation and the breakdown of unreacted ethoxy groups. A degradation step between 250 and 450°C was associated with the decomposition of amide groups into ammonia and water, as well as the breakdown of quaternary ammonium groups [39]. The copolymer nanocomposite demonstrated slightly reduced thermal stability compared with the homopolymer nanocomposite due to the presence of BuMA, which reduces the overall stability of the polymer backbone. Beyond 350°C, additional weight loss was attributed to the decomposition of residual organic fragments and polymer dehydroxylation, ultimately forming carbonaceous char residues alongside the NPs [40]. These weight loss patterns provide further evidence of successful surface functionalization and polymer integration onto MgO NPs.

**Figure 5.**
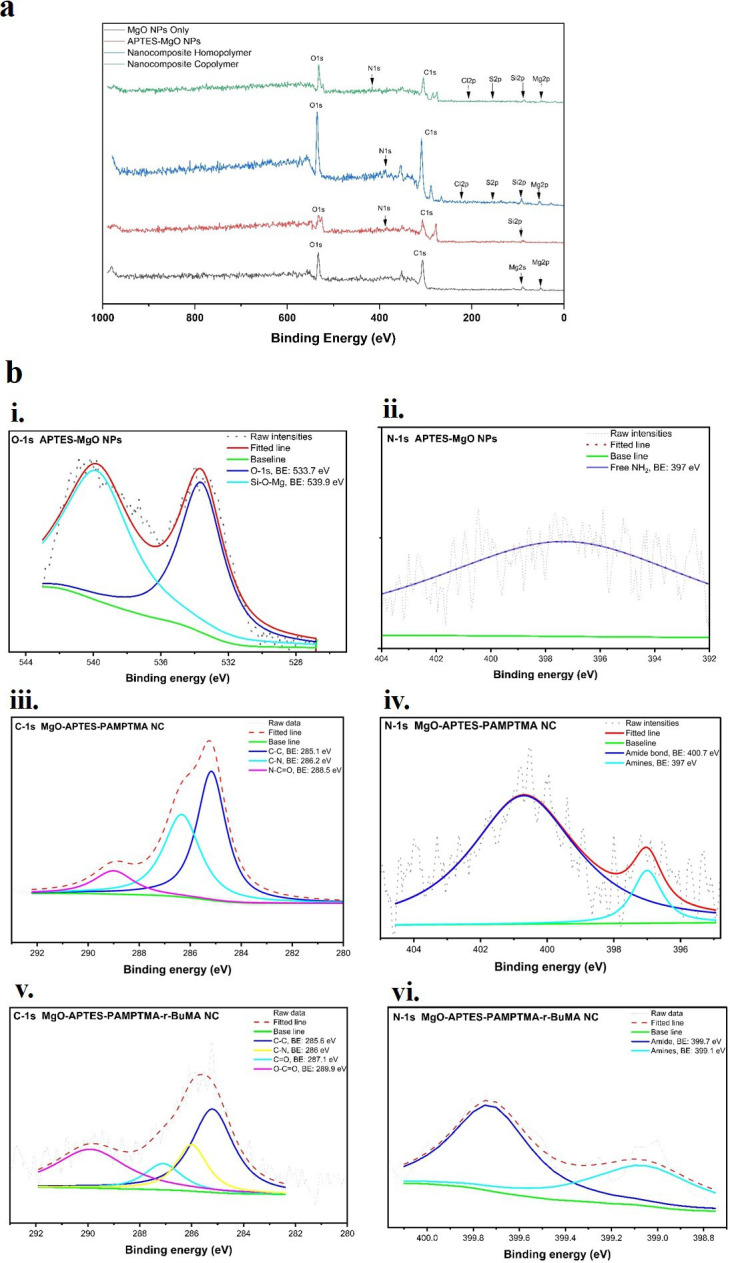
(a) XPS survey spectrum of MgO NPs, APTES-MgO NPs and NCs; (b) high-resolution XPS spectra of (i) O_1*s*_ APTES-MgO NPs, (ii) N_1*s*_ APTES-MgO NPs, (iii) C_1*s*_ NC homopolymer, (iv) N_1*s*_ NC homopolymer, (v) C_1*s*_ NC copolymer and (vi) N_1*s*_ NC copolymer.

XPS was utilized to confirm the surface composition and functionalization of MgO NPs, APTES-functionalized NPs and NCs. Figure 5a illustrates the wide survey spectra for MgO NPs, which revealed characteristic peaks for Mg_2*p*_ (approx. 50.5 eV), Mg_2*s*_ (approx. 90 eV) and O_1*s*_ (approx. 530 eV), consistent with the cubic lattice of MgO. A minor shoulder peak at approximately 532.5 eV corresponded to surface hydroxyl groups and adsorbed water. Additionally, a peak at approximately 300.8 eV was attributed to adventitious carbon contamination, confirming the chemical purity of MgO NPs [41]. APTES functionalization introduced new peaks in the survey spectra, including N_1*s*_ (approx. 398 eV), Si_2*p*_ (approx. 102 eV), C_1*s*_ (approx. 285 eV) and O_1*s*_ (approx. 532 eV). The N_1*s*_ (approx. 398 eV) and Si_2*p*_ (approx. 102 eV) peaks validated the presence of silane coupling, while the C_1_*_s_* peak significantly increased due to the carbon atoms in APTES and any residual adventitious carbon. High-resolution O_1*s*_ scans (532 eV) indicated Mg–O–Si bonding, and changes in N_1*s*_ binding energy (399.3 eV) reflected the amine group coupling (figure 5b). These results confirm the successful APTES functionalization of MgO NPs, consistent with observations by Rabin *et al.* [42]. The XPS survey spectra of NCs revealed additional peaks, including Cl_2*p*_ (approx. 200 eV), and significant enhancement of N_1*s*_ (approx. 400 eV) and C_1*s*_ (approx. 285 eV), confirming the incorporation of PAMPTMA and PAMPTMA-*r*-BuMA polymers. High-resolution N_1*s*_ scans of NC homopolymer exhibited an additional peak of N_1*s*_ with primary amine from APTES using high-resolution scans with binding energy at approximately 400.1 eV, indicating the amide bond formation (figure 5b). The high-resolution C_1*s*_ scans exhibited peaks for C–C (approx. 285.1 eV), C–N (approx. 286.2 eV) and C=O (approx. 288.5 eV) bonds. NC-copolymer scans displayed similar peaks, with enhanced N_1*s*_ (399.7 eV), Cl_2*p*_ (200.1 eV) and C_1*s*_ (285.2 eV) peaks, but included an additional O–C=O peak (approx. 289.9 eV) from the BuMA ester moiety. Slight shifts in binding energies were attributed to the chemical environment influenced by BuMA. These findings validate the successful polymer grafting onto APTES-functionalized MgO NPs, aligning with the work of Lin *et al.* [43].

DLS and zeta potential measurements were conducted to evaluate the hydrodynamic size distribution, dispersibility and surface charges of MgO NPs, APTES-functionalized NPs and NCs. Table 3 summarizes the findings. The hydrodynamic size of commercial MgO NPs was 494 ± 121 nm, larger than the manufacturer-reported size due to aggregation in aqueous media. APTES functionalization increased the size to 761 ± 166 nm, attributed to the added functional layer and enhanced particle interactions, resulting in a larger hydrodynamic radius and some degree of aggregation [44]. Further increases were observed for NCs, with the homopolymer-grafted NC measuring 818 ± 122 nm and the copolymer-grafted NC reaching 1062 ± 136 nm. The extended polymer chains and hydrophobic BuMA groups in the copolymer contributed to these size increases, reflecting the influence of particle–solvent interactions. Zeta potential measurements revealed significant changes in surface charge. Bare MgO NPs exhibited a negative zeta potential of −4.4 mV due to hydroxide ion (OH⁻) adsorption. APTES-modified NPs showed a shift to +6 mV, attributed to protonated amine groups (NH_3_^+^). Homopolymer-grafted NCs had the highest zeta potential (+20 mV), driven by positively charged quaternary ammonium groups in PAMPTMA. Copolymer-grafted NCs showed a zeta potential of +15 mV, slightly lower due to partial shielding by hydrophobic BuMA units. The enhanced zeta potential values for both NCs suggest improved colloidal stability and dispersibility, supported by steric hindrance from the extended polymer chains [40].

**Table 3.** Hydrodynamic size, PDI and zeta potential of MgO NPs, APTES-MgO NPs and NCs.

samples	particle size (nm)	ζ (mV)	PDI
MgO NPs only	494 ± 121	−4.4 ± 1.8	0.4
APTES-MgO NPs	761 ± 166	+6 ± 0.6	0.5
nanocomposite homopolymer	818 ± 122	+20 ± 0.3	0.3
nanocomposite copolymer	1062 ± 136	+15 ± 0.2	0.2

A qualitative ninhydrin test was used to confirm the presence of accessible amine groups on MgO NPs, APTES-functionalized NPs and NCs (electronic supplementary material, figure S4). APTES-modified MgO NPs produced an intense purple colour, indicating accessible primary amines on the surface. In contrast, NCs displayed only faint coloration, attributed to a minimal fraction of unreacted or partially exposed amine groups. The limited reactivity of NCs is expected, as the polymers contain quaternary ammonium groups, which are undetectable by the ninhydrin test.

### 3.3. *In vitro* cytotoxicity determination

#### 3.3.1. 3-(4,5-Dimethylthiazol-2-yl)-2,5-diphenyltetrazolium bromide assay

**Figure 6.**
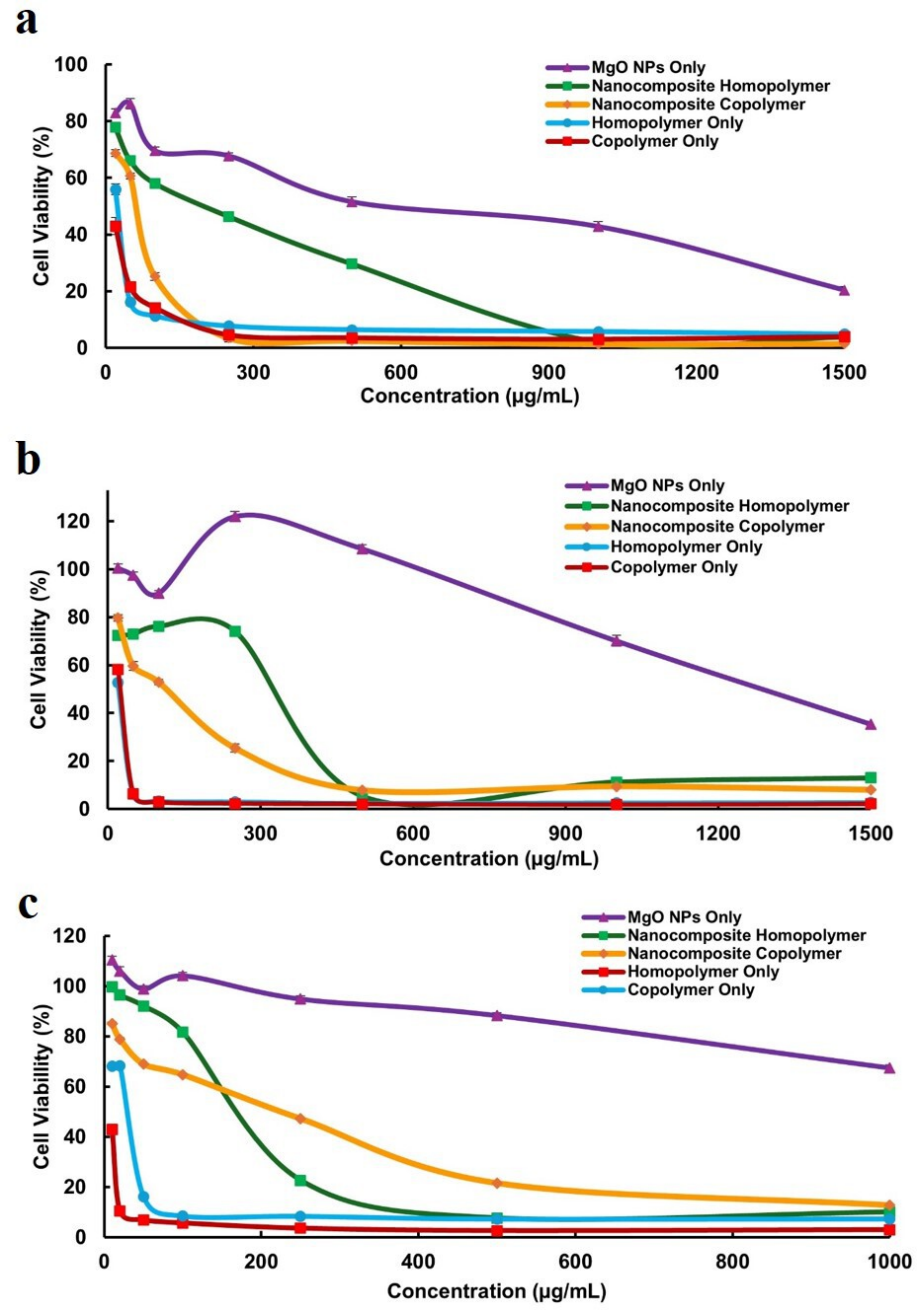
MTT cytotoxicity assay of MgO NPs, nanocomposite-homopolymer, nanocomposite-copolymer, PAMPTMA homopolymer and PAMPTMA-*r*-BuMA copolymer against (a) A-549 cancer cells, (b) Colon-26 cancer cells and (c) HDF normal cells.

The cytotoxic effects of MgO NPs, APTES-functionalized MgO NPs and NCs were evaluated against A-549, Colon-26 and HDF cell lines using the MTT assay. The cytotoxicity was first assessed in cancer cell lines (A-549 and Colon-26) and later extended to normal HDF cells for comparative analysis. Bare MgO NPs demonstrated moderate cytotoxicity at higher concentrations (greater than 250 µg ml^−1^ for A-549 and greater than 1000 µg ml^−1^ for Colon-26), with half-maximal inhibitory concentrations (IC_50_) values of 588 and 1288 µg ml^−1^, respectively (figure 6a,b; table 4). The cytotoxicity of MgO NPs is primarily mediated by the generation of ROS and the release of Mg²^+^ ions, which damage cellular components, including DNA, proteins and lipids. These effects are amplified by smaller particle sizes and acidic conditions, leading to oxidative stress, cellular dysfunction and eventual cell death [45]. However, the high IC_50_ values indicate the limited potency of MgO NPs alone in killing cancer cells, necessitating further modifications for enhanced efficacy. To improve cytotoxicity, two phases of evaluation were conducted: first, for synthesized cationic polymers and, subsequently, for MgO-based NCs incorporating these polymers.

**Table 4.** *In vitro* half-maximal inhibitory concentrations (IC_50_) and selectivity index (SI) of NPs, cationic polymers and their NCs against cancer and normal cell lines.

samples	IC_50_ (µg ml^−1^)			SI	
	A-549	Colon-26	HDF	SI_A-549_	SI_Colon-26_
MgO NPs only	588	1288	1398	2.38	1.09
homopolymer only	25	22	9	0.36	0.41
copolymer only	19	25	31	1.63	1.24
nanocomposite homopolymer	202	338	180	0.90	0.53
nanocomposite copolymer	64	115	226	3.53	1.97

The synthesized cationic polymers demonstrated significantly enhanced cytotoxicity against cancer cells compared with MgO NPs (figure 6a,b). PAMPTMA (homopolymer) exhibited IC_50_ values of 24.5 and 22 µg ml^−1^ for A-549 and Colon-26 cells, respectively. Meanwhile, the PAMPTMA-*r*-BuMA (copolymer) showed IC_50_ values of 19 and 25 µg ml^−1^ (table 4). The increased cytotoxicity of the homopolymer is attributed to its higher molecular weight and longer chain length, which enable stronger electrostatic interactions with negatively charged cancer cell membranes. However, the copolymer displayed even greater cytotoxicity, probably due to the inclusion of the hydrophobic BuMA group. After the initial electrostatic attraction between the positively charged polymer and negatively charged cancer cell membranes, the hydrophobic domain of the copolymer integrates into the lipid bilayer, disrupting its structure. This disruption causes leakage of intracellular components, leading to cell death [32]. The integration of both cationic charges and hydrophobic moieties is thus critical for designing effective anti-cancer polymers.

When polymers were grafted on to MgO NPs to form NCs, their cytotoxicity against cancer cell lines increased compared with bare MgO NPs. The NC-homopolymer exhibited IC_50_ values of 202 and 338 µg ml^−1^ for A-549 and Colon-26 cells, respectively, while the NC-copolymer demonstrated significantly lower IC_50_ values of 64 and 115 µg ml^−1^ (figure 6a,b; table 4). The enhanced cytotoxicity of NCs can be attributed to the combined effects of the NPs and the cationic polymers. The NPs contribute to ROS production and Mg²^+^ ion release [45], while the polymers disrupt cancer cell membranes through electrostatic interactions and hydrophobic domain integration [32]. This combined action disrupts the membrane, increases oxidative stress and causes leakage of vital cellular components, ultimately inducing cell death. The NC-copolymer exhibited a threefold improvement (A-549 IC_50_: 64 µg ml^−1^; Colon-26 IC_50_: 115 µg ml^−1^) in efficacy compared with the NC-homopolymer (A-549 IC_50_: 202 µg ml^−1^; Colon-26 IC_50_: 338 µg ml^−1^). This enhanced performance is attributed to the hydrophobicity of the BuMA component, which increases cellular uptake and disrupts intracellular processes more effectively. These results highlight the enhanced anti-cancer efficacy of cationic polymer-grafted MgO NPs, particularly the NC-copolymer, demonstrating their potential as a drug-free nanoplatform for cancer therapy.

**Figure 7.**
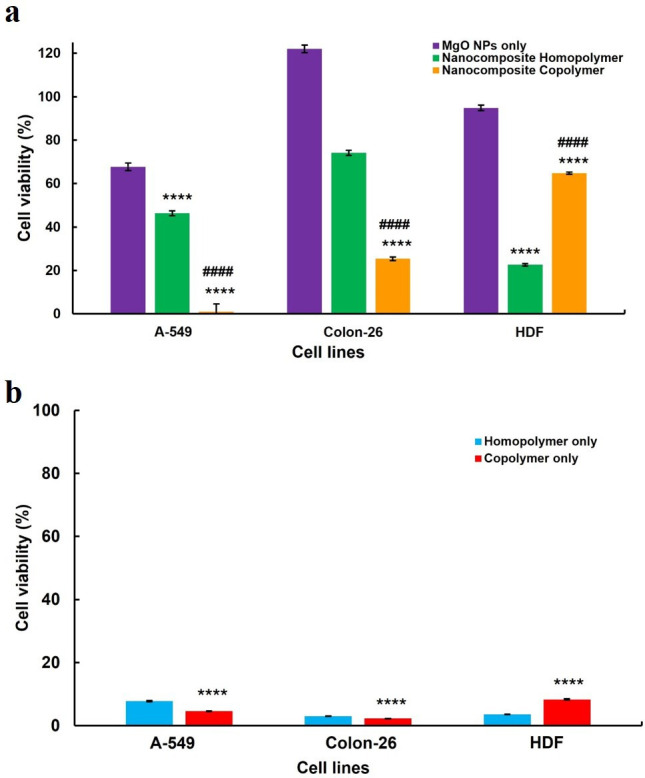
Comparison of cytotoxicity of (a) NPs versus NCs and (b) homopolymer versus copolymer for all cell lines at 250 µg ml^−1^. Error bars indicate standard mean deviation. Statistical analysis was performed using two ANOVA Tukey’s multiple comparison tests (*n* = 3), ****p < 0.0001. NPs versus NCs and homopolymer versus copolymer are represented by *, whereas NC-homopolymer versus NC-copolymer is represented by #.

To further assess the therapeutic potential of the synthesized NCs, cytotoxicity and selectivity studies were extended to evaluate their effects on HDF normal cells (figure 6c; table 4). A key consideration in anti-cancer therapy is achieving selective toxicity toward cancer cells while minimizing harm to healthy tissues. To quantify this selectivity, the selectivity index (SI) was determined by comparing IC₅₀ values between cancer (A-549, Colon-26) and normal (HDF) cells. A higher SI value signifies a greater therapeutic window, highlighting the ability of a treatment to target cancer cells effectively while sparing normal cells. MgO NPs exhibited minimal cytotoxicity, with an IC_50_ value of 1398 µg ml^−1^. This low cytotoxicity is probably due to robust antioxidant defences, efficient DNA repair mechanisms, reduced cellular uptake and extracellular matrix protection in HDF cells [46]. The SI values of bare MgO NPs were relatively low, with SI of 2.38 (A-549) and 1.09 (Colon-26), indicating minimal preferential toxicity toward cancer cells (table 4). In contrast, both PAMPTMA homopolymer and PAMPTMA-*r*-BuMA copolymer also showed higher cytotoxicity in HDF cells, with IC_50_ values of 9 and 31 µg ml^−1^, respectively. The increased cytotoxicity is attributed to electrostatic interactions with negatively charged components of the cell membrane and non-specific cellular uptake. While HDF cells do not exhibit strongly negative surface charges as cancer cells, they still possess some negative charges due to glycoproteins and glycolipids on their membranes [47]. These charges facilitate electrostatic interactions with the positively charged homopolymer, leading to stronger membrane destabilization. The reduced cytotoxicity of the copolymer compared with the homopolymer may result from the inclusion of the hydrophobic BuMA co-monomer, which partially mitigates these effects by shielding the polymer’s positive charges (figure 7b). The SI analysis further clarifies the cytotoxicity profile. The PAMPTMA homopolymer displayed the lowest SI values (0.36 for A-549 and 0.41 for Colon-26), suggesting potent cytotoxicity but with considerable off-target effects on HDF cells. Conversely, the copolymer (PAMPTMA-*r*-BuMA) demonstrated improved selectivity (SI: 1.63 for A-549 and 1.24 for Colon-26), suggesting that the inclusion of the hydrophobic BuMA moiety helped control non-specific interactions while retaining anti-cancer potency (table 4). Upon conjugation of cationic polymers with MgO NPs to form NCs, the cytotoxicity in normal HDF cells was significantly reduced. The NC-homopolymer exhibited an IC_50_ value of 180 µg ml^−1^, while the NC-copolymer displayed an IC_50_ value of 226 µg ml^−1^. The NC-homopolymer exhibited SI values of 0.89 (A-549) and 0.53 (Colon-26), while the NC-copolymer demonstrated the highest selectivity, with SI values of 3.53 (A-549) and 1.97 (Colon-26) (table 4). This enhanced selectivity is attributed to the synergistic effects of polymer-induced membrane disruption and MgO-mediated oxidative stress, which preferentially target cancer cells while reducing interactions with normal cells. These findings suggest that the MgO NPs conjugation mitigates the cytotoxic effects of cationic polymers while retaining strong anti-cancer activity (figure 7a). The pronounced selective cytotoxicity of NCs, characterized by potent activity against cancer cells while sparing normal cells, underscores their potential as promising agents for next-generation anti-cancer therapies. Future studies should focus on improving tumour selectivity through targeted functionalization strategies, such as ligand conjugation or surface modifications to enhance cancer cell recognition. Additionally, comprehensive *in vivo* investigations are necessary to validate their therapeutic efficacy, assess biodistribution and ensure biocompatibility. Addressing these aspects could facilitate the translation of NC-based therapies into future clinical applications, offering a more effective and safer alternative to conventional cancer treatments.

## 4. Conclusion

This study presents the development of cationic polymer-grafted MgO NCs as promising drug-free anti-cancer agents, leveraging the synergistic effects of polymeric charge interactions and the cytotoxic properties of metal oxide NPs. By targeting negatively charged cancer cell membranes, the cationic homopolymer (PAMPTMA) exhibited significant anti-cancer activity through electrostatic interactions, while the hydrophobic copolymer (PAMPTMA-*r*-BuMA) enhanced membrane disruption and permeability, leading to cell lysis. The homopolymer and copolymer displayed potent cytotoxicity against A-549 and Colon-26 cancer cells (IC_50_: 25 and 19 µg ml^−1^; 22 and 25 µg ml^−1^, respectively) but also showed higher toxicity toward normal HDF cells (IC_50_: 9 and 31 µg ml^−1^). In comparison, bare MgO NPs exhibited limited cytotoxicity (IC_50_: 588 µg ml^−1^ for A-549; 1288 µg ml^−1^ for Colon-26; 1398 µg ml^−1^ for HDF), probably due to ROS generation and Mg²^+^ ion release. Cationic polymers grafted on functionalized MgO NPs demonstrated significantly enhanced anti-cancer activity, with lower IC_50_ values against cancer cells (202 and 64 µg ml^−1^ for A-549; 338 and 115 µg ml^−1^ for Colon-26). These NCs exhibited reduced cytotoxicity to normal HDF cells (IC_50_: 180 and 226 µg ml^−1^), suggesting improved selectivity. Among the synthesized NCs, the MgO-APTES-PAMPTMA-*r*-BuMA copolymer displayed the highest selectivity and anti-cancer efficacy, attributed to enhanced ROS production, membrane permeabilization and disruption. This research highlights the potential of cationic polymer-grafted MgO NCs as effective drug-free anti-cancer candidates with reduced off-target toxicity. Future studies should focus on optimizing formulations, *in vivo* validation and exploring the mechanistic pathways to maximize therapeutic efficacy and safety. Additionally, modifying the polymer backbone, tuning molecular weight or adjusting nanoparticle size could further refine selectivity, improve biocompatibility and maximize the clinical potential of these NCs. The findings advance nanomedicine by providing a novel approach to bio-nanomaterial-based drug-free anti-cancer therapeutics.

## Data Availability

The data supporting this article has been included as part of the electronic supplementary material [48].
